# Determining oncology nurses’ awareness of cardio-oncology in Türkiye: A descriptive study

**DOI:** 10.1016/j.apjon.2026.100936

**Published:** 2026-03-12

**Authors:** Yasemin Kalkan Uğurlu, Sevda Türen, Ezgi Karaçam, Geraldine Lee

**Affiliations:** a Faculty of Health Sciences, Department of Nursing, Ordu University, Ordu, Türkiye; b Brookfield Health Sciences Complex, Catherine McAuley School of Nursing & Midwifery, University College Cork, Cork, Ireland; c Independent Researcher, Istanbul, Türkiye; d Dr Sadi Konuk Training and Research Hospital, İstanbul, Türkiye; e Istanbul University–Cerrahpaşa, Postgraduate Education Institute, Istanbul, Türkiye

**Keywords:** Cardio-oncology, Cardiotoxicity, Education, Learning needs assessment, Survey

## Abstract

**Objective:**

The aim of this study was to explore oncology nurses' awareness of cardio-oncology and cancer-related cardiotoxicity.

**Methods:**

This descriptive study was conducted with 152 oncology nurses between April 2025 and October 2025. Data were collected using the *Cardio-Oncology Awareness Questionnaire for Nurses* which was developed by the authors for this study. Descriptive statistics (frequency, percentage, mean, and standard deviation) were used to analyze the data.

**Results:**

Only 15% (*n* = 23) of the participants had received education on cardio-oncology and cancer-related cardiotoxicity and the majority reported acquiring their knowledge primarily from colleagues (63%). Participants expressed limited confidence in recognizing cancer-related cardiotoxicity (*n* = 66, 43%), while the majority (*n* = 147, 97%) indicated a need for further education on cancer therapy–related cardiotoxicity. The educational topics identified were cardiovascular risk factors, basic electrocardiogram (ECG) interpretation and arrhythmias, cardioprotective agents, and cardiovascular risk assessment during cancer survivorship.”

**Conclusions:**

The findings revealed a lack of knowledge and practice regarding cardio-oncology and cardiotoxicity among oncology nurses, with most having not received formal education. With cardio-oncology emerging as an important sub-speciality, there clearly is a need for a structured cardio-oncology education in Türkiye.

## Introduction

Globally, especially in middle to high income countries, cancer remains the prevalent cause of morbidity and mortality.^1^ However, innovations in healthcare have led to earlier diagnosis and the emergence of new treatment approaches for cancer.^2^ Despite these advancements, numerous cardiac side effects (known as cardiotoxicity) emerged following antineoplastic treatments. For example, anthracyclines may cause left ventricular dysfunction and heart failure, while targeted therapies may lead to arrhythmia, hypertension, heart failure, thromboembolic events, and pericardial effusion. Myocardial dysfunction and heart failure arising after cancer treatment are among the most serious cardiotoxicities.^3^ Additionally, coronary artery disease, hypertension, thromboembolism, peripheral vascular disease, stroke, pulmonary hypertension, heart valve disease, QTc interval prolongation, arrhythmias, and pericarditis have been observed.^3^^,^^4^ Preventing cancer-related cardiotoxicity is a key part of treatment. It also ensures long-term survival and quality of life after cancer.^5^^,^^6^

Nurses are the healthcare professionals who interact most closely with patients and are often the first to notice signs or symptoms of toxicity. Oncology nurses play a key role in the administration of antineoplastic treatments, particularly in the early assessment and effective management of cardiovascular risks.^3^^,^^7^ Therefore, oncology nurses should be aware of the cardiotoxic effects that may occur in patients undergoing cancer treatment (electrocardiogram [ECG] changes, arrhythmia, chest pain, dyspnoea, palpitations, changes in blood pressure) and, when these effects occur, should closely monitor cardiac function and take appropriate action. In addition to the signs and symptoms of cardiac toxicity, they should also be familiar with cardiac diagnostic tests such as endocardial biopsy, cardiac biomarkers and echocardiography.^3^^,^^4^

Despite their critical roles in the early diagnosis and management of cardiotoxicity, oncology nurses' knowledge and awareness in the field of cardio-oncology remain limited.^8^^,^^9^ A systematic review from 2015 acknowledged the lack of high-level evidence for the detection and management of cancer related cardiotoxicity. The inability of nurses to recognize early signs and symptoms of cardiotoxicity in a timely manner may lead to delays in treatment, negatively affecting prognosis and potentially contributing to disease progression.

The existing literature has highlighted nurses’ lack of knowledge regarding the monitoring and management of cardio-oncological complications, which poses a direct threat to the quality of care and patient safety.^8^^,^^10^ Moreover, traditional care models are often inadequate in simultaneously and holistically addressing both oncological and cardiological needs of cancer patients.^11^^,^^12^ Personalized and integrated post-cancer care models are needed to address these gaps. Multidisciplinary survivorship care plans can improve patient safety, enhance long-term outcomes, and reduce cancer-related cardiotoxicity.^13^

The absence of cardio-oncology content in nursing curricula limits evidence-based practice and reduces nurses’ effectiveness. According to data collected from ten different countries via a survey developed in collaboration with members of the International Cardio-Oncology Society (ICOS) Nursing Research Group, 88% of the participating oncology nurses (*n* = 268 out of 305) reported that they had not received any formal training in cardio-oncology^8^ The need for a core curriculum for physicians has previously been noted.^14^

Therefore, we conducted an online survey to explore oncology nurses' awareness of cardio-oncology and cancer-related cardiotoxicity from a Turkish perspective. The main objectives were to describe oncology nurses' knowledge-based awareness, assessment practices, and the current use of cardio-oncology practices in clinical settings, and to identify educational needs.

## Methods

### Study design

This descriptive study was conducted with 152 participants between April 2025 and October 2025.

### Population and sample

**Fig. 1:**
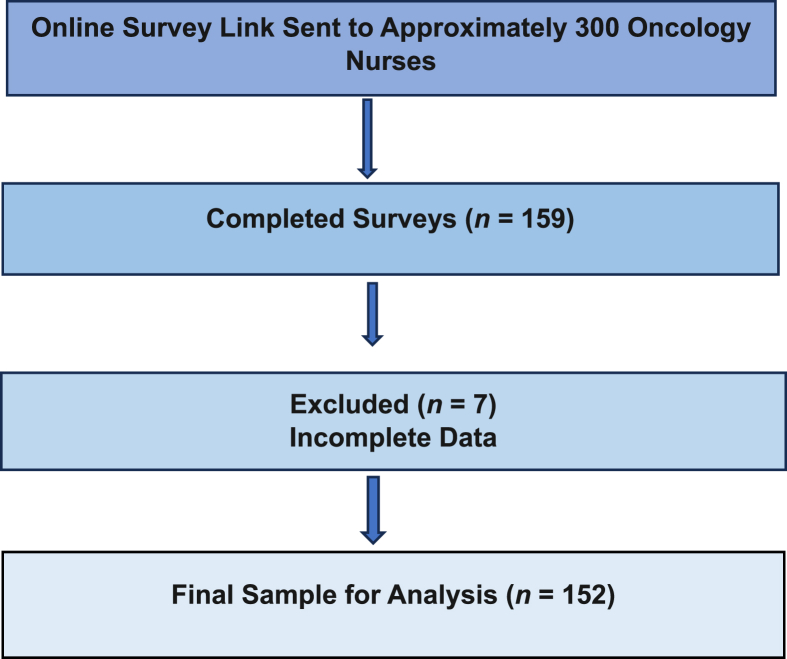
Participant flow chart.

The study included oncology nurses from various regions of Türkiye who met the predetermined inclusion criteria. Inclusion criteria were being an oncology nurse with at least six months of experience, voluntary participation, and having digital access. Participants were recruited using a snowball sampling method via an online Google questionnaire. The online survey link was disseminated to approximately 300 oncology nurses through professional networks and snowball sampling. A total of 159 participants completed the questionnaire. Of these, 7 were excluded due to incomplete data, and the final analysis was conducted with 152 participants (Fig. 1).

The sample size and total number of participants were determined based on a relational research design. Due to the lack of sufficient information on effect size in similar studies—where descriptive statistics are commonly reported—the power analysis was conducted assuming a moderate effect size (effect size: 0.3). Accordingly, the minimum required sample size was calculated as 138 participants for Power: 0.95, β: 0.20, and α: 0.05. Considering potential data loss, the study was completed with 152 participants.

### Study setting

Oncology nurses were invited to participate in the study online. The online Google questionnaire was initially distributed to oncology nurses within the researchers’ professional networks in Türkiye, and they were encouraged to share the forms with other eligible colleagues.

### Data collection tools

Data were collected using the Cardio-Oncology Awareness Questionnaire for Nurses, which was developed by the authors to assess oncology nurses' knowledge-based awareness, assessment practices, and the current use of cardio-oncology practices in clinical settings, and to identify educational needs. The development of the questionnaire was guided by a comprehensive literature review and expert opinions. Current national and international sources on cardio-oncology, cardiotoxicity, and oncology nursing^1^^,^^3^^,^^8^, ^9^, ^10^^,^^15^ were reviewed, and a draft form consisting of 22 items.

The questionnaire was designed as a descriptive data collection form. The content validity of the draft form was evaluated by five experts in the fields of cancer and cardio-oncology (two academic nurses, one cardiologist, one haematologist, and one Clinical Oncology Nurse). Experts were asked to rate the appropriateness of each item on a scale from 1 to 4 (1 = Not appropriate, 2 = Slightly appropriate, 3 = Quite appropriate, 4 = Highly appropriate), and to provide suggestions and corrections for each item. Based on expert evaluations, the Content Validity Index (CVI) for all items was calculated as 0.90. Based on the experts’ feedback, the wording and content of the items were revised; however, the number of items was retained, and the final version of the questionnaire consists of 22 items. The questionnaire consists of three main sections: (i) demographic and clinical characteristics of the participants, (ii) knowledge, assessment, and practices related to cardio-oncology, and (iii) educational needs and priority learning topics in cardio-oncology.

### Ethical considerations

This study was conducted after obtaining approval from the Research Ethics Review Committee of Istanbul Arel University (Approval No. 2025/06, dated 28 February 2025). Nurses who voluntarily agreed to participate were directed to the survey form after reading and approving the online informed consent form. The consent form stated that participants’ responses would be kept confidential and not shared with anyone, that participation in the study was entirely voluntary, and that there was no obligation to take part. All research procedures were conducted in accordance with the principles of the Declaration of Helsinki.

### Data collection

When participants clicked on the survey link, they first encountered a screen containing brief information about the study (including its purpose and inclusion criteria) and an informed consent form. Only those who provided their voluntary consent were granted access to the questionnaire. Completing the survey took approximately 10–15 minutes.

### Data analysis

The findings of the study were analysed using IBM SPSS Statistics version 26.0. Descriptive statistics including frequency, percentage, mean, and standard deviation were used to evaluate the data.^16^

## Results

A total of 152 nurses participated in the study. Many participants were aged between 20 and 30 years (*n* = 82, 54%), female (*n* = 135, 89%), and held a bachelor's degree (*n* = 101, 66%). Most had 0–5 years of general nursing experience (*n* = 56, 37%) and oncology nursing experience (*n* = 116, 76%), worked in oncology ward (*n* = 96, 63%), and reported administering chemotherapy daily (*n* = 82, 54%) (Table 1).

**Table 1:** Demographic and clinical characteristics of respondents (*N* = 152).

Characteristics		*n* (%)
Age (years)	20–30	82 (54)
	31–40	51 (34)
	41–50	19 (13)
Sex	Female	135 (89)
	Male	17 (11)
Highest educational level	High school^a^	9 (6)
	Associate degree	6 (4)
	Bachelor's degree	101 (66)
	Graduate (master's/doctorate)	36 (24)
Nursing experience (years)	0–5	56 (37)
	6–10	47 (31)
	11–20	34 (22)
	21–30	15 (10)
Oncology nursing experience (years)	0–5	116 (76)
	6–10	28 (18)
	11–20	6 (4)
	21–30	2 (1)
Type of oncology workplace	Oncology ward	96 (63)
	Outpatient/clinic/chemotherapy	55 (36)
	Cardio-oncology	1 (1)
Chemotherapy administration frequency	Daily	82 (54)
	Weekly	33 (22)
	Monthly	29 (19)
	Other/as needed	8 (5)

a In Türkiye, nursing education was previously provided both at health vocational high schools and universities. Nurses who graduated from health vocational high schools before the transition to full university-level education are still employed in clinical practice.

Only 15% (*n* = 23) of participants had received education on cancer related cardiotoxicity, primarily from oncology nurses (*n* = 17, 30%). Most reported acquiring information about cardio-oncology from colleagues (*n* = 95, 63%). The majority of participants associated cardiotoxicity with chemotherapy (*n* = 124, 82%). They reported that it could occur during treatment (*n* = 91, 60%), immediately after (*n* = 93, 61%), or years later (*n* = 97, 64%). The most monitored conditions related to cancer related cardiotoxicity were hypertension (*n* = 137, 90%) and arrhythmia (*n* = 131, 86%) (Table 2).

**Table 2:** Knowledge, assessment, and practice in cardio-oncology.

Variable		*n* (%)^a^
I have received cardiotoxicity training (*n* = 152)		
	Yes	23 (15)
	No	129 (85)
If you have received any training on cardiotoxicity, who provided it? (*n* = 57)		
*(Can have more than 1 answer)*	Oncology nurse	17 (30)
	Oncologist	12 (21)
	Official hospital training	8 (14)
	Academic nurse	6 (11)
	Webinar/online training	4 (7)
	Cardiologist	3 (5)
	Cardio-oncology nurse	3 (5)
	Cardiology nurse	2 (4)
	Cardio-oncologist	1 (2)
	Official societies (e.g., ESC or IC-OS)	1 (2)
As nurses caring for oncology patients, what sources do you use to obtain information on cardio-oncology? (*n* = 351)		
*(Can have more than 1 answer)*	Colleagues	95 (63)
	Scientific articles	81 (53)
	Courses/conferences	74 (49)
	Online courses	52 (34)
	National/international guidelines	49 (32)
Which of the following cancer treatments do you think may lead to cardiotoxicity? (*n* = 429)		
*(Can have more than 1 answer)*	Chemotherapy	124 (82)
	Endocrine/Hormonal therapy	44 (29)
	Immunotherapy	86 (57)
	Radiotherapy	90 (59)
	Targeted therapies	64 (42)
	Don't know	21 (14)
When do you think cardiotoxicity is most likely to occur? (*n* = 295)		
*(Can have more than 1 answer)*	During treatment	91 (60)
	Immediately after treatment	93 (61)
	Years after treatment	97 (64)
	Don't know	14 (9)
Which of the following are the main findings that suggest that the patient has developed cardiotoxicity? (*n* = 816)		
*(Can have more than 1 answer)*	ECG changes	129 (85)
	Blood pressure changes	129 (85)
	Heart rate changes	130 (86)
	Fatigue	130 (86)
	Edema	89 (59)
	Dyspnea	97 (64)
	Chest pain	112 (74)
	Don't know	6 (4)
Which conditions related to cardiotoxicity do you monitor? (*n* = 479)		
*(Can have more than 1 answer)*	Heart failure	62 (41)
	Arrhythmia	131 (86)
	Pericardial effusion	32 (21)
	Thromboembolism	70 (46)
	Myocardial ischemia	47 (31)
	Hypertension	137 (90)
What nursing interventions do you think can help prevent cardiotoxicity? (*n* = 992)		
*(Can have more than 1 answer)*	Control infusion rate	140 (92)
	Check drug administration order	135 (89)
	Lifestyle counseling (smoking/alcohol)	103 (68)
	Exercise counseling	94 (62)
	Blood pressure monitoring	135 (89)
	Diet counseling	86 (57)
	Education on medications	107 (70)
	Pre-treatment tests	94 (62)
	Cardiovascular assessment	98 (65)
Which cardio-oncology practices do you implement in your nursing care? (*n* = 538)		
*(Can have more than 1 answer)*	Obtain prior cardiac history	142 (93)
	Assess cardiovascular risk factors	84 (55)
	Evaluate diagnostic tests (ECG, Echo, labs)	83 (55)
	Referral for regular heart check-ups	70 (46)
	Patient/family education on CV complications	88 (58)
	Post-treatment follow-up programs	71 (47)
Do you assess the cardiovascular effects of cancer treatments in your patients? (*n* = 152)		
	Yes	132 (87)
	No	20 (13)
Are you able to recognize potential cardiotoxic effects in your patients? (*n* = 152)		
	Yes	66 (43)
	Partially	66 (43)
	No	20 (13)
Do you think there is a need for more education and training in cardio-oncology nursing? (*n* = 152)		
	Yes	147 (97)
	No	5 (3)

CV, Cardiovascular system; ECG, Electrocardiogram; ESC, European Society of Cardiology; IC-OS, International Cardio-Oncology Society.

a Percentages are calculated based on the number of nurses who responded to each question (*n* = 152). More than one answer could be selected per question.

Preventive measures that oncology nurses reported implementing included infusion rate control (*n* = 140, 92%), checking drug administration orders (*n* = 135, 89%), monitoring blood pressure (*n* = 135, 89%), education on medications (*n* = 107, 70%), lifestyle counselling (*n* = 103, 68%), and exercise counselling (*n* = 94, 62%). Additionally, most participants reported performing cardio-oncology nursing practices such as obtaining prior cardiac history (*n* = 142, 93%), assessing cardiovascular risk factors (*n* = 84, 55%), and patient/family education on cardiovascular complications (*n* = 88, 58%). While 87% of nurses stated that they assess the cardiovascular effects of cancer treatments, only 43% stated that they are able to recognize signs of cancer-related cardiotoxicity. Nearly all participants (*n* = 147, 97%) expressed a need for further education in cardio-oncology nursing (Table 2).

Cardio-Oncology topics identified as priority areas by respondents are presented in Table 3.

**Table 3:** Cardio-oncology topics identified as priority areas by respondents.

Topics		*n* (%)
Cardiology	Cardiovascular risk factors	123 (81)
	Basic ECG interpretation and arrhythmias	116 (76)
	Cardiac medications	109 (72)
	Acute coronary syndromes	72 (47)
Cancer treatments	Cardioprotective agents	112 (74)
	Targeted therapies	95 (63)
	Radiotherapy	86 (57)
	VEGF inhibitors	81 (53)
	Anthracyclines	75 (49)
	Immune checkpoint inhibitors	75 (49)
	Endocrine agents	59 (39)
	EGFR agents	57 (38)
	CAR-T cell therapies	52 (34)
Cardiovascular toxicities	Blood pressure changes	127 (84)
	Heart failure	95 (63)
	Myocardial infarction	95 (63)
	Thromboembolism	95 (63)
	Pericardial effusion/tamponade	71 (47)
	Peripheral vascular disease	70 (46)
	Endocarditis/myocarditis	69 (45)
	Cardiomyopathy	63 (41)
Cancer survivorship	Cardiovascular risk assessment	139 (91)
	Healthy nutrition	100 (66)
	Rehabilitation in cancer patients	95 (63)
	Caregiver involvement in survivorship care	90 (59)
	Supportive/palliative care	87 (57)

ECG, electrocardiogram; VEGF, vascular endothelial growth factor; EGFR, epidermal growth factor receptor; CAR-T, chimeric antigen receptor T-cell. Percentages are calculated based on the number of nurses who responded to each question (*n* = 152). More than one answer could be selected per question.

The most frequently reported topics in the field of cardiology were cardiovascular risk factors (*n* = 123, 81%), cardioprotective agents related to cancer treatments (*n* = 112, 74%), blood pressure changes for cardiovascular toxicities (*n* = 127, 84%), and cardiovascular risk assessment in the context of the lives of cancer survivors (*n* = 139, 91%) as the primary educational needs.

## Discussion

To the best of our knowledge, this is the first survey that explored nurses’ knowledge of cardio-oncology in Türkiye. The survey highlighted the lack of cardio-oncology nurses with only one participant working in the speciality. Contrary to this, Fadol et al.^4^ reported in their international survey that 65% of nurses worked in the cardio-oncology department. This difference in numbers highlights that cardio-oncology is still an emerging field in Türkiye, and nurses have limited experience in this area. Furthermore, only a small number of participants had received formal education on cardio-oncology, with most acquiring knowledge from colleagues. This finding underscores the lack of structured education and the tendency to rely on peer-based learning. Similarly, Fadol et al.^4^ reported low numbers of nurses who had formal training in cardio-oncology, and primarily information was via colleagues or online resources. These results indicate that access to formal education in cardio-oncology is limited both nationally and internationally. This finding is not unexpected when new therapies are introduced.^17^

While many participants correctly identified chemotherapy as a cause of cancer related cardiotoxicity, awareness was lower regarding endocrine/hormonal therapies and targeted therapies. These findings suggest that the full spectrum of cardiotoxicity risks is not yet widely recognized among nurses, especially since the cardiac side effects of endocrine and targeted therapies have only been emphasized in the literature over the past five years.^5^^,^^17^^,^^18^ However, the field of cancer therapies is rapidly evolving and the number of cancer related cardiovascular conditions that require evaluation and management is growing.^19^

Participants demonstrated awareness of both acute (60%–61%) and late-stage risks (64%) associated with cancer-related cardiotoxicity, a finding consistent with the literature indicating that cancer-related cardiotoxicity may develop during treatment or years later.^18^ Furthermore, nurses demonstrated awareness of parameters such as fatigue in identifying ECG (85%), blood pressure (85%), and heart rate (86%) changes, consistent with the current literature.^8^ However, the low awareness among nurses of symptoms such as oedema and dyspnoea is a clinically concerning finding. These symptoms should be considered not only as general side effects of treatment in cancer patients but also as early warning signs (red flags) of serious cardiovascular complications such as heart failure and advanced cardiotoxicity.^8^ Overlooking dyspnoea and oedema can delay cardiotoxicity recognition and worsen patient outcomes.^3^^,^^8^ Nurses should be educated to evaluate these symptoms and promptly inform the healthcare team.

Nurses are typically the first healthcare professionals to intervene in early changes in clinical conditions.^12^ For the early diagnosis of cardiotoxicity symptoms, nurses not only need to be knowledgeable but also confident in their professional competence.^4^^,^^5^ However, less than half of the stated that they are able to recognise cardiotoxicity symptoms, and moderate awareness of cardiovascular complications was identified in cardiovascular risk assessment and patient/family education. Nurses have theoretical knowledge but often struggle to apply it in practice. Almost all participants requested more cardio-oncology education, confirming the literature's call for structured education programs.^9^^,^^20^

In the nursing profession, assessing the patient and providing education according to their needs regarding how to manage their treatment process and care is fundamental. Knowledgeable and educated nurses trained with an approved curriculum will undoubtedly improve patient care.^21^^,^^22^ The IC-OS Nursing Research and Education committees are currently in the process of developing a core curriculum for a Cardio-Oncology Nursing Education programme.^8^ Kolbus et al.^23^ conducted a study with cardiology nurses, implementing a cardio-oncology training program and evaluating its impact on nurses' perceived self-efficacy in managing cancer treatment-related cardiotoxicity. The program was found to be effective in enhancing self-efficacy and emphasized the importance of allocating resources for cardio-oncology education. In this context, our study explored the specific educational needs of oncology nurses and identified priority areas. Participants reported a strong need for training in cardiovascular risk factors, basic ECG interpretation and arrhythmias, cardiac medications, cardioprotective agents, and targeted therapies. These findings highlight the necessity of developing structured, cardio-oncology education programs and integrating them into clinical practice to enhance nurses’ knowledge and competencies.

Initiatives for nursing education in the field of cardio-oncology in Türkiye have begun to increase in recent years.^3^ However, it is observed that education in this field is still not provided within a structured and standardised curriculum. The CardioOncology Project Group, operating within the Turkish Society of Cardiology (TSC), is an important initiative aimed at raising awareness about the cardiotoxicity of cancer treatments.^24^ In this context, the regular bulletins published by the project group summarise current scientific evidence on the cardiovascular side effects of different types of cancer and the agents used in these treatments. Indeed, the latest bulletin, published in 2025, dealt in detail with the effects of prostate cancer and the agents used in its treatment on cardiovascular diseases.^25^

The resources available on the TSC's the CardioOncology Project Group which systematically present the cardiovascular side effects of oncological treatment agents used in clinical practice, provide clinicians with guidance on which agents may cause which cardiotoxic effects.^26^ However, it is evident that these valuable resources are largely physician-focused, with limited structured education programmes and competency-based practices for nurses.

To address nurses' educational needs, it is essential to first develop an appropriate and validated educational curriculum. The curriculum can be integrated into the undergraduate nursing program, for nurses working in the field, in-service education programs, courses and certificate programs can be implemented.^27^ However, rather than one-time education, it is crucial to provide practical, interactive, and continuous education along with written materials aimed at enhancing nurses’ clinical competencies.^21^ Internationally, The International Cardio Oncology Society in conjunction with MD Anderson have recently developed an online Cardio Oncology Nurse Education programme which can be accessed by nurses worldwide and may assist in addressing the current knowledge deficit in cancer related cardiotoxicity. These programs may be particularly suitable for Turkish nurses with sufficient English language skills, and directing suitable nurses to such programs can help address existing knowledge gaps and support competency development in recognizing and managing cancer-related cardiotoxicity.

### Strengths and limitations

A strength of the study was that most study participants were clinical nurses and primarily in oncology. Several limitations should be acknowledged. Firstly, the data was collected online using a self-reported questionnaire, which may lead to inconsistencies between the information participants actually reported and their clinical practices, potentially affecting the reliability of the data. Secondly, participant selection via a Google-based online survey may have created limited control over selection bias and who accessed the survey, potentially under-representing oncology nurses with limited access to digital technology. Therefore, the findings should be interpreted with caution in terms of generalizability. Thirdly, the Cardio-Oncology Awareness Survey for Nurses was developed based on relevant literature and reviewed by experts. Content validity is supported by a Content Validity Index (CVI) of 0.90 for all items, indicating strong expert consensus. As the instrument was designed as a descriptive data collection form and does not produce total or subscale scores, statistical validation procedures such as factor analysis, which should be considered when interpreting the results, were not applied.

## Conclusions

With cardio-oncology emerging as an important sub-speciality, there clearly is a need for a structured cardio-oncology education in Türkiye. Future research using qualitative or mixed-method approaches is needed to better understand nurses’ knowledge, experience, and educational needs.

## CRediT authorship contribution statement

**Yasemin Kalkan Uğurlu:** Conceptualization, Methodology, Software, Writing - Original draft preparation. **Sevda Türen:** Conceptualization, Methodology, Reviewing and Editing. **Ezgi Karaçam:** Visualization, Investigation, Software, Writing. **Geraldine Lee:** Software, Writing - Reviewing and Editing, Supervision. All authors have read and approved the final manuscript.

## Ethics statement

This study was conducted after obtaining approval from the Research Ethics Review Committee of Istanbul Arel University (Approval No. 2025/06, dated 28 February 2025). All research procedures were conducted in accordance with the principles of the Declaration of Helsinki.

## Data availability statement

The data sets produced and/or analysed during this study may be obtained from the corresponding author upon reasonable request.

## Declaration of generative AI and AI-assisted technologies in the writing process

During the preparation of this article, the authors used artificial intelligence tools for language translation support. The authors carefully reviewed and edited the resulting output and are fully responsible for the content of the article. The final version of the article has also been professionally edited.

## Funding

No funding.

## Declaration of competing interest

The authors declare no conflict of interest. The authors declare no conflict of interest. The corresponding author, Prof. Geraldine Lee, is an editorial board member of *Asia–Pacific Journal of Oncology Nursing*. The article was subject to the journal's standard procedures, with peer review handled independently of Prof. Lee and their research groups.
